# Supplementary material for: A Potential Anthelmintic Phytopharmacological Source of Origanum vulgare (L.) Essential Oil against Gastrointestinal Nematodes of Sheep
Source: Animals (Basel). 2022 Dec 22;13(1):45. doi: 10.3390/ani13010045 (PMC9817997; doi:10.3390/ani13010045)
Supplement: Supplementary file 1 [file animals-13-00045-s001.zip › animals-2029929-supplementary.pdf]

Table S1. Impact of *Origanum vulgare* essential oil on the haematological parameters in tested animals – in total from both examined farms.

Parameters	Reference Values	Day	*Origanum vulgare* EO	Albendazole, Control (+)	Sunflower oil, Control (-)
WBC (K/uL)	4.0 - 12.0	0.	8.21 ± 1.8	9.59 ± 1.6	8.78 ± 2.9
		14.	8.82 ± 2.0	9.45 ± 2.6	8.74 ± 2.3
RBC (M/uL)	8.0 - 16.0	0.	8.1 ± 0.9	7.50 ± 0.5	8.01 ± 1.0
		14.	7.58 ± 1.0	7.66 ± 0.6	7.45 ± 1.1
Hgb (g/dL)	8.0 - 16.0	0.	11.35 ± 1.6	10.43 ± 0.5	11.65 ± 1.8
		14.	11.0 ± 1.6	11.08 ± 0.8	10.96 ± 1.7
Hct (%)	24.0 - 50.0	0.	28.37 ± 4.1	26.41 ± 1.6	29.38 ± 4.7
		14.	26.63 ± 3.6	27.30 ± 1.8	27.35 ± 4.2
MCV (fL)	23.0 - 48.0	0.	35.0 ± 2.1	35.17 ± 1.7	36.75 ± 2.4
		14.	35.08 ± 2.3	35.67 ± 2.0	36.75 ± 1.8
MCH (Pg)	9.0 - 12.0	0.	13.95 ± 0.7	13.94 ± 0.6	14.56 ± 0.8
		14.	14.5 ± 0.8	14.52 ± 0.7	14.73 ± 0.8
MCHC (g/dL)	31.0 - 38.0	0.	39.97 ± 1.5	39.53 ± 1.0	39.69 ± 0.8
		14.	41.26 ± 1.0	40.45 ± 0.7	40.14 ± 0.9
RDW (%)	-	0.	16.11 ± 0.8	15.52 ± 0.5	15.93 ± 0.7
		14.	15.93 ± 0.6	15.77 ± 0.5	15.69 ± 0.8
Pit (K/uL)	250 - 750	0.	763.4 ± 220.3	517.0 ± 119.0	545.1 ± 189.1
		14.	631.2 ± 207.8	468.2 ± 142.0	483.0 ± 207.7
MPV (fL)	-	0.	11.77 ± 1.5	11.34 ± 1.5	11.13 ± 1.3
		14.	11.73 ± 1.8	11.42 ± 1.6	10.78 ± 1.3
Pct (%)	-	0.	0.60 ± 0.1	0.60 ± 0.6	0.58 ± 0.2
		14.	0.60 ± 0.2	0.54 ± 0.2	0.46 ± 0.1
PDW (%)	-	0.	5.98 ± 9.3	5.43 ± 4.5	5.83 ± 8.0
		14.	5.77 ± 8.7	4.59 ± 5.0	9.23 ± 9.6
Lin (K/uL)	-	0.	4.63 ± 1.1	4.94 ± 1.3	4.16 ± 1.1
		14.	4.72 ± 1.3	5.41 ± 2.5	4.73 ± 1.6
Lin (%)	40 - 75%	0.	56.67 ± 7.2	51.83 ± 12.0	52.83 ± 12.3
		14.	55.60 ± 8.8	56.42 ± 17.1	54.17 ± 6.8
Gra (K/uL)	-	0.	3.58 ± 1.1	4.65 ± 1.4	4.09 ± 2.4
		14.	4.09 ± 1.3	4.04 ± 1.7	4.01 ± 1.2
Gra (%)	-	0.	43.33 ± 7.2	48.17 ± 12.0	47.17 ± 12.3
		14.	44.40 ± 8.8	43.58 ± 17.1	45.83 ± 6.8

* No statistically significant differences were found when comparing values on D14 compared to D0, nor when comparing values for different groups on the same day (p>0.05).
